# Contesting authority in breastfeeding support: Maternal negotiation of professional and embodied knowledge

**DOI:** 10.1177/13591053251383744

**Published:** 2025-11-04

**Authors:** Christina Severinsen, Mary Breheny, Angelique Reweti

**Affiliations:** 1 Massey University, Palmerston North, Manawatu, New Zealand

**Keywords:** breastfeeding, discourse, medicalisation, motherhood, autonomy, professionalisation, embodiment

## Abstract

Breastfeeding is a physiological process with profound relational, emotional, and cultural meaning, yet postnatal care often reduces it to a technical task. This study examines how mothers in Aotearoa New Zealand navigate breastfeeding support through Well Child services, focussing on tensions between professional expertise and embodied maternal knowledge. Using an online platform, Wāhi Kōrero, 420 anonymous stories were collected in response to the prompt: “Kōrero I wish I could’ve had with the Well Child nurse.” A subset of 149 stories referencing breastfeeding was analysed using thematic and discourse analysis. Findings reveal conflicting discourses: breastfeeding as an embodied, relational practice versus a technical skill requiring correction. Mothers did not reject professional expertise but resisted prescriptive, standardised advice through strategies such as selective disclosure, strategic compliance, and hybrid knowledge use. These findings contribute to feminist critiques of medicalised motherhood and suggest reframing professional expertise as an epistemic partnership that validates both clinical and maternal ways of knowing.

## Background

Feminist scholarship provides critical insights into how breastfeeding is situated within the sociopolitical dynamics of medicalised motherhood. As a physiological process imbued with sociocultural meaning, breastfeeding exemplifies how maternal bodies are subjected to medical surveillance and control (Nisha, 2022). Medicalisation reframes embodied maternal experiences as clinical, fragmenting women’s embodied knowledge and privileging scientific authority over intuitive, relational understandings (Stankovic, 2017; Zaslow, 2012). Feminist theorists highlight how maternal bodies become contested sites where lived experience competes with biomedical discourses about infant nutrition and development (Young, 2020). These frameworks carry unequal power to regulate the maternal experience, making breastfeeding particularly susceptible to disempowering forms of medicalisation. This tension is intensified by disciplinary power in breastfeeding support – the mechanisms through which professional authority shapes maternal behaviour – where mothers are expected to succeed with breastfeeding yet must submit to professional instruction on how to do so “correctly.” Breastfeeding support is never neutral but reflects power relations that constrain autonomy and marginalise embodied knowledge (Young, 2020).

### Professional breastfeeding support

Breastfeeding outcomes are shaped by biological, social, and structural factors, with healthcare support playing a critical role in initiation and duration (Fu et al., 2014). Despite the emphasis on evidence-based, individualised, and culturally responsive postnatal care, research highlights persistent inconsistencies in support provision (Dawson et al., 2019; Dol et al., 2022). The professionalisation of breastfeeding support contributes to these dynamics, as formal training and clinical guidelines increasingly regulate the practice (Hausman, 2003). This establishes a hierarchy where professionals become legitimate experts, while mothers are positioned as laypersons whose embodied experiences can be improved by professional intervention. This mirrors medicalisation processes, reframing maternal care as clinical tasks requiring oversight.

Tensions persist between protocol-driven breastfeeding support and the need for flexible, individualised care. While guidelines can offer consistency, rigid adherence can overlook the nuanced needs of mothers and infants (Lubbe, 2018). The emphasis on standardisation and biomedical authority can compromise maternal agency when support is instructional rather than relational (Chambers et al., 2023; Ryan et al., 2017). While breastfeeding guidelines may emphasise responsive, culturally, family-centred care, implementation often defaults to standardised protocols that prioritise measurable outcomes (Ministry of Health, 2020a, 2020b, 2024; WHO, 2023a, 2023b). Maternal departures from professional advice can elicit disapproval, contributing to self-doubt and disengagement (Severinsen et al., 2024). Support perceived as condescending or directive can feel dismissive, particularly when mothers’ personal, cultural, or contextual circumstances do not align with professional recommendations (Severinsen et al., 2024). Public health framings of breastfeeding as a risk-reduction practice further reinforce narrowly defined best practices and marginalise relational, community-based approaches (Subramani et al., 2023).

Disparities in breastfeeding support along socioeconomic and cultural lines further exacerbate inequities in maternal experience. In Aotearoa New Zealand, Māori, and Pacific mothers report encountering less accessible, less culturally safe, and more surveillance-oriented approaches to support, reflecting systemic inequities in maternal healthcare (Ministry of Health, 2021). Young mothers, in particular, are framed as inexperienced or irresponsible, and their competence is routinely questioned (Breheny and Stephens, 2007; Severinsen et al., 2024). While some marginalised mothers actively resist these stigmas by asserting their knowledge and autonomy, the absence of affirming, strengths-based support models remains a barrier to equitable care (Severinsen et al., 2024).

Despite extensive research on breastfeeding support, tensions between professional authority and maternal expertise remain underexamined. These tensions are likely to be particularly encountered within services offered postpartum to new mothers, where health professionals play a prominent role in both supporting mothers and monitoring health outcomes. This study addresses this gap by exploring how mothers navigate interactions with Well Child nurses and how these encounters shape breastfeeding experiences. By centring maternal perspectives, this research offers an in-depth analysis of the structural and interpersonal dynamics that underpin breastfeeding support, contributing insights with implications for policy, professional training, and the development of equity-focussed, relationship-based models of care.

## Method

This study explores mothers’ stories of breastfeeding support within Well Child Tamariki Ora home-visiting services in Aotearoa New Zealand. The stories were shared anonymously through the online platform Wāhi Kōrero (www.wahikorero.co.nz; see Severinsen et al., 2025) for methodological details). The project was promoted through social media and Action Station, a community advocacy organisation, inviting people to share their stories in response to the prompt: “Kōrero (conversations) I wish I could’ve had with the Well Child nurse.” Submissions were open from August to September 2021. A total of 420 stories were submitted, primarily by women (average age 34 years). Of the 61% who reported ethnicity, 19% identified as Māori, 3% as Pacific, 81% as NZ European Pākehā, and 6% as other ethnicities. This broadly reflects national demographics, although Pacific Peoples were underrepresented. Participants could read submitted stories, and the platform was actively moderated to ensure anonymity and safety. Ethical approval was granted by the Massey University Human Ethics Committee (SOB 21/30), and all identifying details were removed before analysis. Participants explicitly consented to research use of their stories when submitting to the platform, with clear information provided about the study’s purpose and data usage (Severinsen et al., 2025).

From the complete data set, we identified all stories that referenced breastfeeding (*n* = 149), which formed the subset for this analysis. This focussed subset provided accounts of how professional authority, maternal expertise, and structural conditions shape breastfeeding support experiences.

### Analytic approach

The Wāhi Kōrero research team brings together expertise in critical public health, health psychology and Māori health, enabling multiple analytic perspectives while maintaining a focus on addressing inequities in health outcomes. For this analysis, we used a combined thematic and discourse analytic approach. Braun and Clarke’s (2006, 2013) six-phase framework guided the thematic analysis, including familiarisation, coding, and theme refinement. The process was iterative and overlapping, moving between semantic coding (explicit meanings) and latent interpretation (underlying assumptions and discursive constructions). In parallel, a discourse analysis lens examined how language constructed professional authority, maternal expertise, and institutional norms. We analysed how mothers positioned themselves in relation to healthcare providers, how authority was established or contested, and how accounts reflected or resisted dominant discourses about breastfeeding. This included attention to how language framed breastfeeding as a skill, a biological process, or a site of struggle. The analysis proceeded iteratively, moving between individual accounts and broader discursive patterns, with regular team discussions to refine emerging interpretations. The identification of conflicting discourses – breastfeeding as embodied practice versus breastfeeding as a learned skill – developed through this iterative process. This approach captured mothers’ experiences of breastfeeding support and understood these experiences as shaped by power relations inherent in interactions between mothers and health professionals.

Using a discourse analysis approach, we viewed language as constitutive of meaning, focussing on how meanings are constructed through available discursive resources (Parker, 2014; Willig, 2022). In this analysis, the text reflects the reported breastfeeding experiences that mothers chose to share, which are shaped by and articulated through broader social discourses. This integration allowed us to acknowledge mothers’ reported experiences while critically examining how these experiences are situated within and shaped by medicalised frameworks of maternal care. To ensure analytical rigour, two researchers independently coded the data. Initial codes were developed inductively through close reading of all the data, and discrepancies were resolved through discussion and iterative refinement. Particular attention was paid to identifying and illustrating the key features of each discursive construction to demonstrate how each discourse was made convincing. Verbatim quotes were selected to illustrate key themes, to ensure participants’ accounts remained central to the analysis, and to ensure the credibility of the analysis. Combining thematic analysis with discourse analysis enabled a nuanced analysis of maternal experiences and the discursive construction of breastfeeding support within child health visiting services.

## Findings

Mothers’ experiences of breastfeeding support within Well Child services were influenced by two discursive constructions: breastfeeding as an embodied practice and breastfeeding as professional expertise. These discourses are constitutive, shaping subjectivities, structuring power relations, and enabling or constraining action and resistance. Their interaction produces contested sites where different knowledge forms are negotiated and debated. We examine each discourse separately, exploring how they position mothers and professionals, construct risk and responsibility, and shape maternal subjectivity and agency before analysing how mothers strategically navigate these competing discursive constructions.

### Breastfeeding as embodied practice

#### The discursive construction of embodied knowledge

The first discursive construction positions breastfeeding as an intuitive, relational experience, guided by maternal knowledge and capacity to respond to infant needs. Mothers articulate this by emphasising intuition, responsiveness, and embodied knowing, constructing breastfeeding as developing naturally through attunement. Accounts used affective terms like “felt so wrong” or “knew in my heart,” when contrasting embodied responses with professional directives. Intuition was constructed not only as valid but essential for sustaining breastfeeding when professional advice misaligned with maternal experience.

Part of embodied breastfeeding knowledge involved understanding bodily responses and sensations as valuable information. Mothers described pain, tiredness, exhaustion, or dreading feeds as key information shaping their understanding of what was working. From an embodied discursive construction, such corporeal information is valuable:

I had my first baby at 19, she never lost any birth weight and was a ‘good’ feeder. They told me it was painful because it was a new experience, she was getting upset at feeding because she was ‘cat napping’ which was bad, and I needed to hold off feeding her for a minimum of 4 hours, even if she was crying and to try stop the crying any other way than feeding, walk in the pram, rocking, putting her in the bassinet and walking away. She was three months and had a lip tie ’causing bad latch, I received no support other than judgment for disagreeing that I would continue to demand feed my exclusively breast feed baby. My well child [nurse] made me feel like I was doing wrong by not listening to her, it was very judgmental vibes. (Participant 140)

In this discursive construction, pain as normal is refuted and reframed as evidence of feeding issues. The directive to delay feeding regardless of the baby’s cues is overridden in favour of maternal responsiveness through demand feeding and listening to the baby’s cues.

#### The temporal dimension of maternal expertise

The second distinctive feature is its temporal framing; breastfeeding knowledge is viewed as developmental and cumulative. Maternal expertise is constructed as evolving through experience:

I wish I could have discussed how much breastfeeding hurt sometimes. . . But the only time I tried to bring that up I was told I should just put my 3 month old baby on formula. . . I wish I could have talked about a lot of things, things that 16 years and 4 children later I now know are absolutely normal. . . (Participant 172)

Experiences previously pathologised are reframed as “absolutely normal,” and advice to cease breastfeeding is reinterpreted as silencing maternal struggles. This evolving knowledge enables critical engagement with professional advice across successive children. Growing experiential knowledge becomes epistemic capital for contesting professional claims about later children. Confidence develops through successive mothering experiences.

#### Relational dimensions of breastfeeding

A third distinctive discursive thread constructs breastfeeding as fundamentally a relational practice encompassing emotional connection, comfort, and attunement. Mothers assert the legitimacy of breastfeeding’s affective, interpersonal dimensions. Breastfeeding is constructed as a responsive practice shaped by unique mother-infant dynamics:

I wish that I could have talked to my nurse about co-sleeping, extended breastfeeding. . . All the things that I knew in my heart were right, but I felt so judged. . . After extensive research of my own. . . I’ve realised that my nurse was not at all an expert in normal infant sleep, breastfeeding or attachment theory. . . once I gave up trying to change her and embraced the stages we were in/trusted myself and my daughter then our family thrived. (Participant 773)

The phrase “knew in my heart” locates authoritative knowledge within the maternal body, while “trusted myself and my daughter” positions breastfeeding as a reciprocal relationship. Babies are attributed agency and constructed as sophisticated communicators whose needs are best interpreted through maternal responsiveness:

I felt so unsupported & disempowered by Plunket with my first daughter. . . I was stressed out because she was a ‘low percentile’ in weight and it was suggested that she wasn’t getting enough milk. . . I had to schlep up to the Plunket rooms weekly to get her weighed. . . By the time my second bubba came around I was much more confident. . . All in all a better experience to trust my own instincts. (Participant 664)

Clinical practices often privilege weight measurement, yet participants affirm maternal intuition as a more reliable indicator of wellbeing. This marks a discursive shift from disempowerment to reclaiming embodied authority. It generates a complex maternal subject – intuitive yet contested – who is simultaneously knowledgeable through embodied connection and vulnerable to invalidation by professional authority.

Because knowledge is contextual and relational, family knowledge is positioned as authoritative. This knowledge arises only through engagement with family preferences and circumstances:

I felt judged for decisions I made about feeding my 9 month old during the night, being told she doesn’t need feeding during the night. I also felt there was no understanding of our family situation. . . I had finally been feeling confident with our routine. . . but my confidence was shattered within one conversation with someone who didn’t know us but felt they had the right and the knowledge to tell me what I was doing wrong. . . I’m doing my best. . . to back myself and what we are doing, as we know our baby best and what works for our wee family. (Participant 784)

This account elevated situated family knowledge over decontextualised professional advice. The assertion “we know our baby best” establishes experiential, family-specific knowledge as more legitimate than external expertise that fails to reflect lived realities.

This relational construction emerges powerfully when mothers directly challenge professional advice that attempts to separate nutrition from comfort:

I also struggled to breastfeed. . . They were supportive but then my daughter turned one and they told me she no longer needs breastmilk that’s it’s just for comfort! Misinformation again! I quoted the WHO. . . How can Plunket be so uneducated? As if ‘just for comfort’ is a bad thing. . .?! We are raising emotionally intelligent humans who need connection/comfort etc. (Participant 358)

This account contests the devaluation inherent in the phrase “just for comfort,” which linguistically subordinates emotional nurturing to nutrition. By linking breastfeeding to the development of “emotionally intelligent humans,” this participant repositions the relational aspects of breastfeeding as developmentally significant.

Responsiveness emerges as central to this relational construction, with mothers articulating how breastfeeding enables attunement to infants’ needs:

I was so overwhelmed. . . When I was honest about my struggle to others who were meant to support me I was told I was the problem because I was anxious and the solution was to stop breastfeeding/give a bottle/separate from my baby. This was so against what I felt to be right. . . There was no cultural context for simply responding to your child and meeting their needs consistently. It was as if a mothers instinct and intuition were value-less and silly. . . (Participant 594)

Professional advice is positioned in direct opposition to maternal knowledge. The assertion that the advice lacked “cultural context” highlights how relational approaches exist outside professional frameworks for breastfeeding support. Within health professional settings, maternal instinct is positioned as “value-less and silly,” revealing the systematic delegitimisation of embodied knowledge in favour of separation-oriented approaches.

This discourse generates distinctive subject positions for both mothers and infants. Mothers are positioned as emotionally attuned caregivers, whose responsiveness constitutes expertise transcending technical knowledge. Babies are constructed as communicative agents with legitimate needs for comfort and connection. Together, the mother-infant dyad becomes a mutually constitutive and authoritative source of knowledge about appropriate breastfeeding practices.

### Breastfeeding as professional expertise: A learned skill discourse

#### The discursive construction of breastfeeding as a technical practice

The second discursive construction frames breastfeeding as a technical skill requiring professional oversight. Authority is located in clinical expertise, scientific evidence, and measurable outcomes. Success is constructed through adherence to prescribed techniques and metrics such as weight gain and feeding schedules. In this construction, breastfeeding is a skill requiring specific instruction and correct technique. This discursive construction positions professional support as evaluative, instructional, and necessary for achieving breastfeeding success.

Breastfeeding is constructed as a skill that can be formally taught by professionals.

I wish my well child nurse would have asked about what I was worried about, what my concerns were or things I wanted help with . . . I wish she was better equipped to teach me how to breastfeed. (Participant 84)

Here, breastfeeding is constructed as a technical skill to be transmitted to the mother by the professional. The expectation of being “taught” positions the mother as a recipient of expertise, reinforcing clinical authority. This framing is further evident in the emphasis on bodily technique. Professional attention to posture, positioning, and “correct” methods reconfigures breastfeeding as a procedural act assessed against standardised norms. Mothers become subjects whose success depends on technical compliance:

When I had my first son, the first Plunket nurse we had seemed quite old skool, like she had a set method. . . As a first time mum I didn’t feel comfortable questioning this ‘expert’. . . I remember her telling me I needed help with my posture when breastfeeding. I couldn’t make her ‘method’ work, I was really uncomfortable. . . (Participant 182)

Technique becomes both the object of instruction and the metric of maternal competence. The nurse’s authority is constituted through the establishment of “correct” methods and standardised practices that mothers must learn and replicate. Bodily discomfort is interpreted not as feedback requiring adaptation of method, but as evidence of technical failure:

I was lacking a lot of confidence but thanks to my amazing midwife, we had found a position that was comfortable for both me and my baby. . . But when the plunket nurse observed a feed (despite me requesting that she didn’t), her comments made me feel small and incompetent. ‘You’ll never be able to feed him anywhere else if that’s how you feed him’. . . . (Participant 891)

This account demonstrates how the “skill” construction fragments breastfeeding into assessable technical components, privileging standardised techniques over comfort or efficacy. The professional claims both expertise to identify deficiencies and authority to override maternal preferences. This disciplinary regime scrutinises maternal bodies, positioning mothers as novices judged by technical conformity rather than embodied knowledge.

#### Standardisation and measurement

Maternal accounts reveal a discourse that constructs breastfeeding primarily as a measurable, quantifiable process, reconstituted through standardised and visual representations:

With my first child it was about meeting the growth milestones, she just never quite made it and I was left to feel like I wasnt providing enough milk. . . The whole visit was regimented, questioning felt like cross examining and I always arrived ready to feel judged as a failure. . . In every other way she was a thriving baby. . . The fixating on a graph and defined set of norms meant no conversation between the Wellchild Nurse and myself were easy, free from judgement and allowed me to have a reciprocity of dialogue. . . (Participant 157)

Breastfeeding is equated with functional milk production, defining success through quantifiable outputs measured against population norms. Mothers are positioned as milk producers whose success can be objectively verified. The discourse collapses breastfeeding to providing enough milk, framed by a production-consumption equation. While offering clarity through concrete measurements and establishing clear intervention parameters, this construction narrows the professional gaze. Growth charts and percentile measurements function as standardisation technologies, establishing normative ideals against which infant health and maternal competency are judged. Indicators of wellbeing beyond weight gain are subordinate. Professional engagement thus prioritises measurement over holistic assessment, even when babies are “thriving” in other ways.

The regulation of feeding schedules contributes to the discursive construction of breastfeeding as a professional practice. Mothers who feed on demand are asked to quantify the frequency of feeds and are advised to conform to standardised routines:

When asked how often my daughter breastfed I answered honestly that she was fed on demand. She could not accept this and pushed and pushed me to define how frequently this was. . . This was my second baby who was several months old and in the 90th percentile. I had no concerns about her weight gain. . . She suggested that I limit her feeds to four hourly. Having already breastfed my eldest daughter on demand for two years, I wasn’t looking for any advice about this. . . (Participant 341)

Despite evidence of optimal growth, the inability to numerically specify breastfeeding frequency is framed as non-compliance. Maternal expertise, derived from prior successful breastfeeding, is overridden by professional guidance that privileges regularity and measurability over responsive care.

#### Risk and safety in professional discourse

Professional authority in breastfeeding contexts is enacted through risk invocation, where certain practices are framed as potentially harmful and others as safe. Professional control is legitimised by invoking possible future harms, governing maternal behaviour through fear, responsibility, and moral obligation. Risk discourse displaces harm into the future, constructing present practices as consequential for long-term outcomes. Maternal breastfeeding thus becomes a high-stakes decision:

I also regret not questioning on her advising me to stop (breast) feeding my 3 month old so much and to give him a dummy to shush him instead of my breast. Her reasons being that if I over feed him now it’ll lead to childhood obesity issues. (Participant 645)

Responsive breastfeeding is reframed as “overfeeding,” with potential consequences of “childhood obesity” used to justify professional intervention. Risk discourse constructs a binary of “safe” versus “risky” practices and mobilises developmental threats to override maternal preferences:

My first born, now 4 years, was a slow grower. I was told to formula feed him which I didn’t want to do. I was told his brain wouldn’t develop properly and it was probably because I was back at work so early. I know now that my boy was just a slow grower because my girl is even smaller! I never received any lactation support, dietary advice, any of the basics. I look back and now I can see he had a dairy intolerance because his sister has the same. (Participant 96)

The stark warning – “his brain wouldn’t develop properly” – operates as a powerful affective lever, compelling compliance through fear. While centring on formula feeding, this illustrates how breastfeeding as a professional skill deploys risk rhetoric to displace maternal knowledge and reassert authority. Attributing harm to maternal employment adds a moralising dimension, suggesting maternal choices are actively harmful. Risk discourse operates through affective intensities, particularly fear, that discipline maternal behaviour. These accounts illustrate how a fear of future harm secures compliance.

Within these accounts, mothers are positioned as requiring professional intervention and surveillance to mitigate risks to their children’s safety. Practices such as comfort-feeding or co-sleeping are constructed as warranting monitoring, casting professionals as expert risk managers who identify danger and prescribe safe practices. Babies are constructed as inherently vulnerable and in need of protection. Mothers are simultaneously held accountable for long-term outcomes, yet constructed as lacking expertise to manage risk independently. Risk management thus becomes a central organising principle shaping maternal practices and professional-mother interactions.

### Contested authority and discursive tensions

#### Challenging the evidence base of professional expertise

One way mothers rejected professional advice was by highlighting its lack of scientific basis. Mothers strategically appropriated scientific authority to challenge professional guidance within the dominant biomedical paradigm, reframing themselves as informed evaluators rather than passive recipients of advice. Mothers cited sources, such as the World Health Organisation, to assert alternative interpretations of breastfeeding guidelines:

Misinformation again! I quoted the WHO that recommend breastfeeding for two years or more. How can Plunket be so uneducated? As if ‘just for comfort’ is a bad thing. . .?! (Participant 358).

By referencing the World Health Organisation, this mother appropriates scientific authority to counter professional advice, recasting it as “misinformation” and elevating her own as better aligned with current evidence. Her rhetorical move (“How can Plunket be so uneducated?”) inverts the conventional expert-layperson dynamic, positioning the professional as lacking knowledge and herself as scientifically literate. This strategy constructs mothers as critical consumers of healthcare, assessing advice against external evidence. Mothers expressed disappointment in professionals’ lack of evidence-based knowledge about breastfeeding, implying that professional legitimacy is contingent upon staying current with research. By listing multiple areas where “current research” diverges from received advice, mothers establish themselves as scientifically literate subjects capable of evaluating the quality and scope of professional knowledge. Temporal markers (“20 years or more”) become credibility markers, as mothers evaluated professionals based on alignment with current research. Mothers appropriate scientific discourse to challenge professional advice, positioning themselves not as intuitive actors outside of science but as more current and comprehensive interpreters of scientific knowledge than the professionals. This disrupts the presumed exclusivity of professional expertise while remaining anchored in the scientific paradigm.

Educational credentials offer some mothers the confidence to resist or critique professional advice:

She had lots of other totally incorrect advice so I lost confidence in her . . . I ended up just going through and lying entirely to avoid awkward conversations. I have a bachelors in health sciences so felt confident enough to guess my way through parent hood. I went to plunket I felt as if I needed to fit into their box, to nod and agree, to say what they wanted me to say. There was no partnership. I didn’t feel I could go to them for advice and when I did it was totally incorrect advice. I felt quite let down. (Participant 74)

Formal education provides a resource for evaluating advice and resisting professional authority, positioning the mother within a knowledge system that enables critique. She identifies the advice as “totally incorrect” and ceases relying on professionals. However, this knowledge generates tension: she remains compelled to “fit into their box” and conceal her disagreement. Even for mothers with institutional capital, resistance extends only to the professional advice received; mothers are still positioned as needing to comply with the monitoring inherent in this discursive construction. Mothers must engage in strategic navigation – complying, resisting, withdrawing – based on available resources and constraints. Young, less experienced mothers without educational credentials are particularly vulnerable to judgement and dismissal, while older, more experienced, or educated mothers are more able to evaluate and resist inappropriate or harmful advice.

When mothers challenge professional advice by citing scientific sources, they paradoxically reinforce the underlying premise of the professional skill discourse: that legitimate knowledge must be scientifically validated. This strategic reconfiguration produces hybrid subjectivities that selectively integrate professional expertise with embodied knowledge, prioritising infant wellbeing and positioning mothers as autonomous actors, capable of evaluating, and questioning professional knowledge.

#### Competing constructions of risk and safety

Across mothers’ accounts, persistent tension emerged between professional and maternal discourses of risk, revealing competing understandings of safety. These divergent frameworks produce contradictory advice and practices, leaving mothers in fraught positions where trust, compliance, and care become contested. A striking feature is the inversion of professional authority, where mothers construct certain health advice not only as unhelpful but actively dangerous. Rather than deferring to professional discourse, they assert their own expertise, grounded in embodied knowledge, peer networks, and emotional intuition:

When my baby was 6 months old and refusing solids my nurse advised me too stop breastfeeding her so that when she gets hungry enough she’ll eat. Luckily I had great support and education through la leche leauge and knew to ignore that but for a new mother who didn’t know better this could have been very dangerous advice. (Participant 696)

Here, the mother positions professional advice as a threat to her baby’s wellbeing, reconstituting this as “very dangerous.” She asserts alternative expertise, such as La Leche League, as a safer and more legitimate source of knowledge. This framing contests the normative assumption that professional guidance necessarily reduces risk, instead foregrounding a maternal discourse that prioritises attunement and relational care, which underpins knowing what advice to follow and to ignore.

Although mothers can construct professional advice as “dangerous,” the uneven power dynamics between mothers and nurses compel mothers to conceal their practices, fearing judgement or reprimand. Concealment becomes a protective strategy for both the child and the mother’s autonomy:

For my first child I used to hide what I was actually doing. . . I slept my child on his side as his tummy was sore after feeding. . . I was too scared to tell her I had made the decision to bottle feed as I couldn’t cope anymore. . . During these times for new mums we need a space to have open and honest communication. . . They aren’t even allowed to teach you about bottle feeding! That is terrible. (Participant 179)

Maternal decisions, grounded in immediate needs, are delegitimised by dominant risk discourse, creating an environment where honesty is foreclosed. Using the example of bottle-feeding, this account illustrates how the construction of non-compliance with professional advice as risky silences mothers. This silencing prevents advice on delegitimised practices, such as bottle-feeding or bed-sharing, from being raised, which may increase rather than reduce harm.

Risk discourses are used to frame mothers as potential threats, necessitating correction through surveillance and standardisation. In contrast, mothers position professionals as the risk, particularly when recommendations seem to dismiss the child’s individuality or the mother’s intuition. These discursive conflicts extend beyond individual practices to broader diagnostic frameworks. Some mothers challenge not only specific advice but the epistemological foundations of professional assessment:

I needed support due to my son waking every 15-20 minutes at night for months. . . instead of my concerns being taken seriously I was told I was ‘starving him’ by almost exclusively breastfeeding. . . I didn’t listen and did my own research- he had a dairy and egg allergy and also had 90% blocked nasal passages. . . A child waking every 15-20 mins at night is NOT NORMAL. . . their recommendations were dangerous. . . thank-goodness I followed my heart. . . (Participant 821)

In this account, professional advice is constructed as misdiagnosis, rooted in a normalising logic that attributes difficulties to maternal practices and fails to account for specific medical conditions. The mother positions breastfeeding and co-sleeping as protective, while characterising recommended “solutions” as negligent and potentially fatal. Maternal intuition (“followed my heart”) is revalorised as critical knowledge, contrasting with perceived mechanistic and depersonalised clinical reasoning. Babies are positioned as standardised bodies requiring uniform care when breastfeeding is seen as a professional skill, and as unique individuals requiring responsive, contextualised parenting when breastfeeding is constructed as an embodied practice. In navigating competing constructions of risk, mothers are not passive recipients of knowledge but active agents reshaping the meaning of safety. Their accounts highlight relational and epistemic ruptures when institutional authority fails to accommodate complexity and difference. These hybrid maternal subjectivities are fluid and adaptive, prioritising relational wellbeing and functional outcomes over technical orthodoxy.

#### The medicalisation of maternal emotions

Another recurring tension in mothers’ accounts is the discursive construction of maternal emotions. While mothers construct emotional experiences as integral to their breastfeeding, professional discourse tends to minimise or pathologise these emotions, treating them as irrelevant to feeding outcomes or as indicators of maternal dysfunction requiring management. This medicalisation repositions affective responses within clinical paradigms, shaping how mothers are perceived and perceive themselves in professional encounters.

A prominent feature of this discursive process is the transformation of emotional distress into a technical problem. Instead of acknowledging maternal emotions as valid, professionals reframe them as evidence of incorrect parenting, shifting focus from the mother’s wellbeing to behavioural correction:

On one of my first visits to Plunket I was showing visible signs of being overwhelmed, over tired, stressed, anxiety etc. . . Instead of referring me for PND help or talking about self care. . . the nurse put a video on her laptop for me to watch and left the room. It showed a mother and baby entering a nursery, mum says goodnight, pats baby and leaves the room and baby magically falls asleep. So I was left feeling like I was doing it all wrong. . . My PND went undiagnosed for 2 more months until I presented with mastitis at my GPs. . . Thankfully my GP listened and I got the help I needed. . . (Participant 704)

Here, visible signs of distress are treated as opportunities for correction. The nurse’s decision to show an idealised video rather than engage with the mother’s emotional state reflects professional logic that attributes distress to technical failures. This reframing compounded the mother’s sense of failure (“I was doing it all wrong”) and delayed the recognition of postnatal depression. Because breastfeeding as a professional skill focuses on technique and milk provision, emotional ambivalence is resolved through the technical solution of formula feeding, positioning emotional expression as evidence of bodily failure rather than as part of the typical maternal experience. In this context, emotional disclosure becomes risky, potentially positioning the mother as unstable, incompetent, or failing. By privileging technical competence and clinical categorisation, professional discourse restricts what can be said, heard, or supported.

#### Reclaiming autonomy and expertise

Rather than internalising professional judgement, many mothers describe strategic acts– ranging from defiance to quiet noncompliance– that resist external authority over their bodies, practices, and identities. They reclaim autonomy and assert expertise in response to professional dismissal or criticism (“just to prove her wrong!”; Participant 373). Others adopt covert strategies, appearing compliant while continuing to trust their own judgement. This resistance allows them to avoid confrontation while preserving autonomy:

My second child was a chubba bubba! She was over the top of the height-weight chart at her 4 month check up. Now I was as surprised as anyone at the measurements, but I was advised to cut out night feeds. As a second time mum I smiled and nodded and changed absolutely nothing, continued on with bed sharing and boobing all night and all day. At our next appointment I received praise for following instructions and helping my baby be healthy. My Plunket nurse basically put my 4month old daughter on a diet to fit a chart, and showed her lack of understanding of normal child growth spurts. (Participant 702)

This account reveals a dual performance: outward compliance conceals a confident reliance on embodied maternal knowledge. Professional praise (“for following instructions”) becomes ironic, underscoring the limits of clinical observation and the spaces mothers claim for independent decision-making. Autonomy is maintained through discretion rather than open defiance. Other mothers resist more openly, refusing to comply with advice they perceive as misaligned with their infant’s needs. Disengagement becomes a decisive challenge to authority – an act of empowerment rather than defeat. By stepping outside the “field of visibility” maintained by professional oversight, mothers contest institutional claims to define legitimate practices. Mothers act with discernment, drawing on multiple ways of knowing to support caregiving aligned with their values and their infant’s needs.

Mothers defended their parenting choices despite repeated judgement, describing being “told off” and “scolded” to highlight the disciplinary nature of professional encounters. These strategies - defiant persistence, strategic compliance, and direct confrontation – reflect a broader renegotiation of maternal subjectivity. These acts reconceptualise breastfeeding as an embodied practice, repositioning mothers as knowledgeable agents and challenging professional discourses that position them as passive recipients of professional advice. Accounts of embodied practice construct maternal identity through lived experience and everyday wisdom, questioning the assumed superiority of clinical knowledge and exposing the contested nature of postnatal care. However, mothers do not reject all professional input. At times, they seek advice to support breastfeeding. However, they actively evaluate, reinterpret, and sometimes reject this advice, exercising discretion in ways that balance it against other evidence: bodily responses, relational knowing, infant responses, and community values.

## Discussion

Mothers’ engagement with breastfeeding support is shaped by two discursive constructions: one that positions breastfeeding as an embodied and relational practice, and another that frames it as a technical, biomedical task requiring professional management. However, our findings reveal these are not oppositional frameworks. Instead, mothers navigate a complex epistemic terrain, drawing on multiple knowledge sources to construct individualised approaches. They enact sophisticated negotiations that integrate evidence-based knowledge with embodied maternal expertise, selectively using clinical insights while resisting standardised approaches that dismiss their lived experiences and observational insights. This “both/and” navigation demonstrates how mothers actively synthesise rather than choose between different ways of knowing.

### Tensions between embodiment and professional expertise

This analysis highlights a fundamental tension in breastfeeding support: the coexistence of discourses that frame breastfeeding both as a relational act and as a technical process requiring expert intervention. This builds on feminist critiques of the medicalisation of motherhood, where processes like childbirth and breastfeeding are reframed as clinical events requiring surveillance and control (Hausman, 2003; Torres, 2014). Our findings align with Burns et al. (2013), who describe a dominant “mining for liquid gold” discourse, where midwives prioritise technical competence and milk transfer over the relational dynamics of feeding. This reflects the technocratic care model identified by Davis-Floyd (2001), where the maternal body is treated as a malfunctioning machine requiring professional management.

In tracing how professional authority is produced and maintained, our study builds on Dykes (2006) description of “disconnected encounters,” where support is governed by standardised protocols that obscure individual needs and contexts. Mothers’ accounts reveal how infants’ developmental variations, feeding patterns, and temperamental differences, and maternal responses, shape breastfeeding practices. However, these individual characteristics are often overlooked when professional support privileges population norms over dyad-specific responses. Breastfeeding is constructed as a skill to be taught, assessed, and corrected, privileging clinical observation over embodied knowing. However, the mothers in our study did not reject expertise entirely; they valued technical support for specific challenges, such as latching and developmental information. What they resisted were the technologies of power– growth charts, feeding schedules, checklists– operating as instruments of surveillance and judgement. This aligns with McLeish et al.’s (2021) findings that while mothers welcome knowledge, they are highly sensitive to its form and tone. Although Torres’s (2014) concept of “medicalising to demedicalise” describes how lactation consultants use clinical authority to reduce intervention, our findings suggest a parallel maternal strategy. Mothers also strategically draw on biomedical language to validate their experiences while resisting the normative expectations often accompanying professional advice. Similarly, Tomori et al. (2016) found mothers resist moralising narratives around stigmatised practices, such as bedsharing or extended breastfeeding, while continuing to engage with biomedical discourse. Our data echoes this pattern: rather than rejecting or complying, mothers enact discursive negotiation, assembling, and reworking knowledge in ways that align with their values and lived realities.

### The disciplinary mechanisms and risk discourse

The disciplinary logics that underpin many breastfeeding interventions align with Foucault’s (1978) notion of biopower: governance through normalisation, self-surveillance, and routine correction rather than coercion. Clinical quantitative parameters used by healthcare professionals, such as percentile charts, developmental milestones, and feeding routines, render mothers and infants visible and governable. As Burns et al. (2013) show, midwifery care often asserts authority by treating the breastfeeding body as clinical equipment, reinforcing expert ownership over maternal function. Subramani (2024) extends this argument by demonstrating how breastfeeding interventions are shaped by cultural, political, and ideological assumptions about maternal responsibility. Certain groups are disproportionately targeted for surveillance and intervention, framed as needing correction rather than support. In this context, even benign deviation from normative expectations is framed as risky, prompting clinical response even when breastfeeding is effective. This reflects Foucault’s (1978) “normalising judgement,” where statistical norms become regulatory benchmarks.

These mechanisms extend beyond physical regulation to produce emotional and moral effects. Lupton (2012) argued that maternal risk discourse often relies on temporal displacement, framing present behaviours in terms of future harm (e.g. linking responsive feeding to later obesity). This displaces attention from current maternal and infant needs, alongside mothers’ experiential observations, fostering what Ryan et al. (2010) describe as the “moral work” of motherhood– a constant negotiation of competing standards under uncertainty and fear. Maternal accounts reveal how disciplinary power operates through self-surveillance; at times, mothers strategically reconfigure their disclosures to avoid judgement and obscure non-compliant practices, such as bed sharing. These dynamics reflect the affective force of risk discourse, which regulates practice and shapes mothers’ self-perceptions as caregivers. McLeish et al. (2021) similarly found that how support is delivered, more than what is said, most often undermines maternal confidence. These disciplinary mechanisms directly generated corresponding resistance strategies: when nurses privileged technical expertise over maternal knowing, mothers developed counter-expertise through research; when growth charts pathologised normal variations, mothers deployed their own evaluative frameworks emphasising holistic thriving over percentiles; and when risk discourse displaced current wellbeing, mothers enacted temporal reframing that prioritised immediate responsive care.

### Practices of resistance and epistemic hybridity

Tensions between embodied knowledge and professional expertise prompted specific forms of resistance. When professional authority dismissed comfort feeding as superfluous, mothers revalued it as developmentally significant. When standardised measurements contradicted maternal perceptions of thriving babies, mothers used embodied observations as counter-evidence, transforming sites of surveillance into opportunities for epistemic assertion. Despite disciplinary power, our findings highlight the subtle but significant ways mothers resist. Drawing on Foucault’s (1988) concept of “practices of freedom,” we noted various resistance strategies, including overt defiance, selective disclosure, passive compliance, and complete disengagement. These actions allowed mothers to maintain autonomy without overtly challenging professional authority. In some cases, mothers’ bodily experiences contradicted clinical assessments, enacting what Butler (1993) terms “corporeal resistance” – where the body itself becomes a site of dissent simply by functioning outside professional expectations.

Importantly, mothers did not reject biomedical knowledge outright. Instead, they engaged in strategic epistemic hybridity, drawing on both experiential and scientific knowledge to construct their own frameworks. The tension itself became productive, generating new maternal subject positions that neither discourse alone could accommodate. This reflects Lupton and Tulloch’s (2002) concept of situated risk rationalities: personalised, context-sensitive evaluations of risk that contrast abstract or statistical accounts. In this hybrid positioning, mothers repurposed expert discourse to assert authority while maintaining the relational and embodied values central to their own definitions of good care. This finding extends the work of Tomori et al. (2016) on counter-narratives built through embodied knowledge and relational trust. It also supports Schmied et al.’s (2011) call to recognise maternal knowledge as legitimate. Our findings suggest that mothers reject the passive position of recipients of professional advice. Instead, they negotiate a position as knowing subjects – critical, selective interpreters of expert advice. Subramani (2024) locates these choices within broader constraints, noting that maternal resistance involves navigating cultural and structural limitations while preserving core values.

### Implications of the research

These findings suggest the need for breastfeeding support models responsive to the epistemic complexity of maternal experience. Mothers are not choosing between expertise and instinct; they seek to integrate technical, embodied, and socially situated experiences in ways that affirm both infant wellbeing and maternal agency. Effective support must move beyond standardised guidance to prioritise relationship-building, responsiveness, and contextual sensitivity. As McLeish et al. (2021) conclude, while mothers value practical help, they resist directive, surveillance-based approaches. We propose reconceptualising professional expertise not as epistemic dominance but as *epistemic partnership*– a collaborative model in which professionals and mothers co-construct care through mutual respect and dialogue. This also entails expanding definitions of success beyond clinical metrics to include maternal satisfaction, confidence, and relational attunement. These findings suggest that mothers also play an active role in shaping these partnerships– engaging strategically, asserting embodied knowledge, and co-producing the meanings of good care. However, at the institutional level, current models often remain invested in standardisation, surveillance, and measurable outcomes (Davis-Floyd, 2022; Dykes, 2006; Ministry of Health, 2020a, 2024; WHO, 2023a, 2023b). Transforming these systems requires reconfiguring metrics of success and restructuring services to support care that is not only evidence-based but relationally and culturally grounded.

### Strengths and limitations

A key strength of this research was the ability of the Wāhi Kōrero platform to capture anonymous accounts that mothers felt unable to share with health professionals. The platform’s asynchronous design minimised researcher influence over the construction of these accounts, providing an opportunity for mothers to describe encounters with child health visitors without fearing judgement (Severinsen et al., 2025). While anonymous data collection facilitated disclosure, it limited the analysis to the features of breastfeeding experiences that participants shared. There is some evidence in the data that these experiences were shaped by social identities such as education and ethnicity; however, the anonymous platform limited our ability to fully explore intersectional analyses regarding the impact of multiple social positions on breastfeeding experiences and resistance strategies. Although the online platform was key to accessing these stories, this also creates the potential for digital exclusion of participants without internet access or digital literacy. Other approaches to data collection that target rural, remote, or digitally excluded communities may be necessary to ensure marginalised mothers have opportunities to share their experiences. The combined thematic-discourse analytic approach proved useful in revealing how experiential patterns were shaped by power dynamics in healthcare interactions. Future research could explore this experiential and professional disjuncture by including professional perspectives on breastfeeding support interactions. Analysing health interactions from the professional vantage point could further inform approaches to strengthen epistemic partnership.

### Conclusion

The challenges mothers face within Well Child services are shaped by broader systemic structures. The tension between breastfeeding as an embodied practice and as a learned skill creates contradictions in how support is provided and received. Mothers often seek help when breastfeeding is difficult, but this support is frequently prescriptive or dismissive of their embodied experience. When services prioritise structured, skill-based interventions over relational, intuitive care, they risk undermining maternal confidence. An integrated, responsive model – one that values both maternal experience and professional expertise – can help bridge these tensions by offering evidence-based support and being attuned to the lived realities of breastfeeding. This study contributes to the breastfeeding literature by identifying systemic and interpersonal barriers to effective support and advocating for care that is relational, responsive, and culturally safe. Professional guidance should function as a facilitative rather than directive force – affirming maternal agency and supporting, rather than constraining, breastfeeding success.
